# Pharmacy practice and clinical pharmacy research in the Middle East: a scoping review of studies from Bahrain, Iraq, Jordan, Kuwait, Lebanon, Oman, Palestine, Qatar, Saudi Arabia, Syria, United Arab Emirates, and Yemen

**DOI:** 10.1186/s40545-022-00434-y

**Published:** 2022-06-08

**Authors:** Daneh Obaid, Faris El-Dahiyat, Zaheer-Ud-Din Babar

**Affiliations:** 1 Clinical Pharmacy Program, College of Pharmacy, Al Ain University, P.O Box 64141, Al Ain, United Arab Emirates; 2 AAU Health and Biomedical Research Center, Al Ain University, Abu Dhabi, United Arab Emirates; 3 Department of Pharmacy, School of Applied Sciences, University of Huddersfield, Huddersfield, HD1 3DH West Yorkshire UK

**Keywords:** Pharmacy practice, Pharmacy practice research, Middle East

## Abstract

**Background:**

Pharmacy practice research publications has increased significantly in the last decade. This is also true for Middle Eastern countries.

**Aims:**

The aim of this study was to document and review pharmacy practice literature in the Middle Eastern Arab countries.

**Materials and methods:**

A scoping review was conducted using PRISMA-ScR guidelines. Medline/PubMed and Scopus were used to screen the articles. All published original research articles concerning any facet of pharmacy practice in 12 Arabic Middle Eastern countries during 2009–2019 were included. A thematic analysis was performed to classify the articles.

**Results:**

Nine hundred and eighty-one articles were included in this study. Eight themes emerged from the selected articles. Medication use was the predominant theme 30.78% (302), followed by pharmacy practice and pharmacist services 22.94% (225), and then pharmacy education and professional development 16.31% (160). The KSA, Jordan, Qatar, and the UAE were the leading countries to publish pharmacy practice research.

**Conclusions:**

Pharmacy practice research is growing and significantly adding to enhance pharmaceutical health services in the Middle East Region. There is a need to develop a research agenda. This will help in enriching the practice, as well as to avoid repetitive ideas.

## Background

Pharmacists, the medication experts, have a dynamic role in the health sector [1]. A remarkable change has endured in pharmacy practice throughout the past decade. The pharmacist role has expanded beyond traditional practice into more clinical patient-centered practice [2–6].

This dynamic shift ascends the necessity of having evidence that evaluates pharmacy services, assesses the need for new services or enhancement of the available services, and specify the benefit and cost of the new advanced pharmacists’ role; which overall emphasize the importance of pharmacy practice research development [7–14].

Pharmacy practice research endorses the value of the potential pharmacist’s role and services [10, 14, 15], generates ideas for new pharmacy services; in addition, it also supports policymakers and regulatory affairs decisions [7, 10, 15, 16].

Up to date, there is no global or universally accepted definition of pharmacy practice or pharmacy practice research. Several studies aimed to figure out a comprehensive definition of pharmacy practice and its disciplines [17, 18]. In 2019, Hasan et al. conducted an exploratory study to define pharmacy practice and it’s research, where several definitions were collected and analyzed. In conclusion, Hasan et al. illustrated the major themes comprising pharmacy practice and pharmacy practice research, which were medicines use, patient-centered care, and health services delivery [18].

In this review, we adopted the International Pharmaceutical Federation (FIP), Pharmacy Practice Research Special Interest Group (2019), pharmacy practice research definition as it is the most recent universal definition. The FIP Definition states: “a component of health services research that emphasizes the impact of the practice of pharmacy on the healthcare systems, medicines use and patient care. It’s scope has expanded over the past 50 years to encompass aspects, such as the clinical, behavioral, economic and humanistic implications of the pharmacy practice as well as practice change and implementation of innovations and new services in routine practice.” [19].

For centuries, Arabs intensively influenced the science and art of pharmacy, which subsequently contributed to the evolution of pharmacy in Europe, and eventually, the rest of the world [20, 21].

Pharmacy education and pharmacy practice in Arabic-speaking Middle Eastern countries continue to evolve. However, there is no published comprehensive review that outlines the overall status of pharmacy practice research in this region. Hence, this paper aimed to provide an inclusive review and explore the gaps in pharmacy practice research.

### Study rationale

To the best of our knowledge obtained from the literature search, no published review covered pharmacy practice research in the Middle East comprehensively; however, few studies have outlined few domains of pharmacy practice research, such as education [22], medication safety [23, 24], pharmacovigilance [25], and pharmacoeconomics [26].

### Study significance

Pharmacy practice research in the Middle East is in the developing stage. Exploring the current status of pharmacy practice research in several practice domains will highlight the drawbacks and help in drawing a roadmap for the upcoming research studies in this region.

### Study aim

This study aims to evaluate the current status of pharmacy practice research in 12 Arab Middle East countries by retrieving related published articles.

### Specific objectives

The study was conducted to:

- Explore, review, and synthesize literature published in the 12 Arab Middle East countries, related to pharmacy practice in its major domains (Community, Hospital, Clinical, Educational, Economics, and Social and Administrative aspects).
- Explore topics and themes covered in the literature.
- Identify the limitations and areas that needed further development.
- Provide suggestions to develop and improve the pharmacy practice research.

## Methods

### Study design

**Fig. 1:**
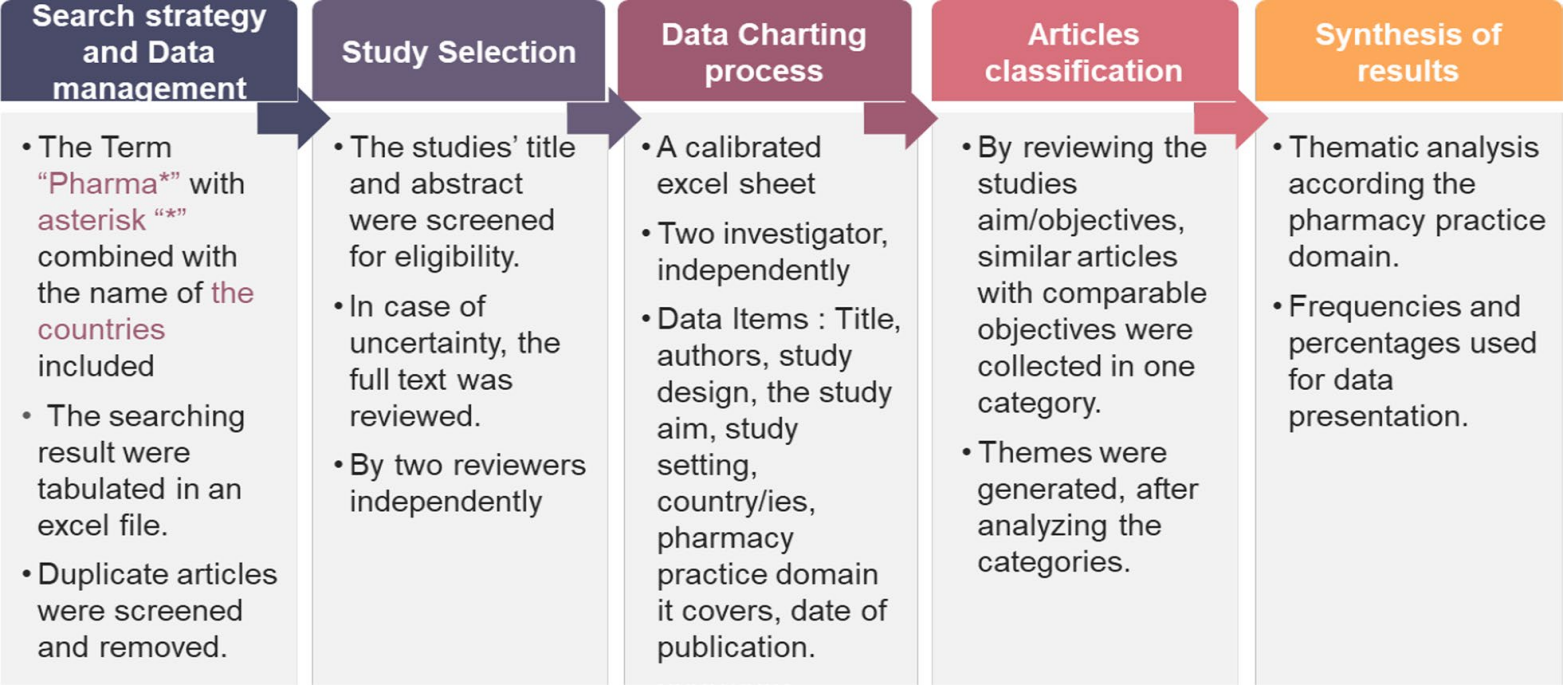
Method steps

A Scoping review using PRISMA-ScR guidelines [27] was conducted to retrieve published literature in the field of pharmacy practice in 12 Arabic Middle East countries. The countries included Bahrain, Iraq, Kingdom of Saudi Arabia, Kuwait, Lebanon, Oman, Palestine, Qatar, Syria, United Arab Emirates, Yemen, and Jordan. The method steps of this study is shown in Fig. 1.

### Eligibility criteria

Literature published in the English language within 10 year interval [Jan. 2009–Dec. 2019] covering pharmacy practice domains in the selected Middle East countries; (Bahrain, Iraq, Kingdom of Saudi Arabia, Kuwait, Lebanon, Oman, Palestine, Qatar, Syria, United Arab Emirates, Yemen, and Jordan), were included in the review. Only original articles were involved excluding non-original articles as: letters, commentaries, systematic reviews, editorials; in addition, studies-related to other pharmacy aspects, such as pharmaceutics and drug formulation, pharmacology studies, pharmaceutical chemistry, and pharmacognosy were excluded.

### Information resources

PRISMA-ScR guidelines for scoping review were followed. The databases were searched from Aug.-2019 till Jan.-2020. The Database included: The Medline database using PubMed and Scopus database. In the search process, “Boolean Operator” rules were employed. The terms used were searched utilizing “AND” to combine the keywords listed and “OR” to remove search duplication where possible.

Results details included; the database, publishing journal, article title, authors, and the publication date. The detailed search strategy conducted is clarified in Table 1.

**Table 1:** Search results details by the database

Database	Searching terms	Searching filters	Searching result	Last searching date
PubMed	Pharma* combined with keywords that specify the name of the countries included (“Gulf cooperation council” OR “Emirates” OR “Saudi Arabia” OR “Oman” OR “Kuwait” OR “Bahrain” OR “Qatar” OR “Yemen” OR “Iraq” OR “Jordan” OR “Syria” OR “Lebanon” OR “Palestine”)	[Title/Abstract] AND (2009:2019)	1337	June-2021
Scopus			2521	June-2021

### Search strategy

A search strategy was developed and implemented. The search was restricted to the English language using the following keywords: “Pharma*” AND (“The Middle East” OR “Gulf cooperation council” OR “Emirates” OR “Saudi Arabia” OR “Oman” OR “Kuwait” OR “Bahrain” OR “Qatar” OR “Yemen” OR “Iraq” OR “Jordan” OR “Syria” OR “Lebanon” OR “Palestine”). The keywords were combined and incorporated in database searches to collect the findings that contain the selected terms in the title or the abstract of the articles published from 1st Jan-2009 till 31st Dec. 2019.

### Studies selection

Duplicate articles were screened and removed. The title and abstract of the studies were screened by two reviewers to test their eligibility and relevancy. The articles were categorized into three groups: included, excluded, and uncertain. In case of uncertainty about an article’s inclusion, the full text was retrieved and reviewed by the two authors/reviewers independently to decide the eligibility state of the article. However, if the conflict remains, assistance from a third expert was acquired. After excluding the irrelevant articles, the eligibility of all included articles was assessed by full-text screening. Articles were excluded for the following reasons; if published in a non-English language, in different practice settings irrelevant to pharmacy practice, conducted in countries other than the ones listed, unoriginal articles, such as comments, letters, case reports, surveys, editorials, review articles, unpublished articles, or articles in-press.

### Data charting process

Two investigators were assigned to extract data from the articles and categorize them according to the pharmacy practice domain it covers, independently. In a calibrated excel sheet, the data was presented based on the following; Title, authors, study design, the study aim, study setting, related countries, pharmacy practice domain it covers, and date of publication. By reviewing the studies’ aim/objectives, similar articles with comparable objectives were collected in one category, and after analyzing the categories; the themes were generated.

### Data items

Title, authors, type of research or study design (research methodology if any), site of study setting (Hospital, Community pharmacy, General pharmacy, Educational Institution, or Public setting), related countries, pharmacy practice domain it covers, date of publication, publishing journal, study aim, are the data that were collected and tabulated from each study. After the extraction of relevant information, a thematic synthesis was undertaken of the study objectives, types, and pharmacy practice domain it covers; to identify themes based on frequencies (No. of source found) for each.

### Synthesis of results

The data were thematically analyzed according to the pharmacy practice domain. Frequencies and percentages were used to present the synthesized results.

## Results

### Study selection

**Fig. 2:**
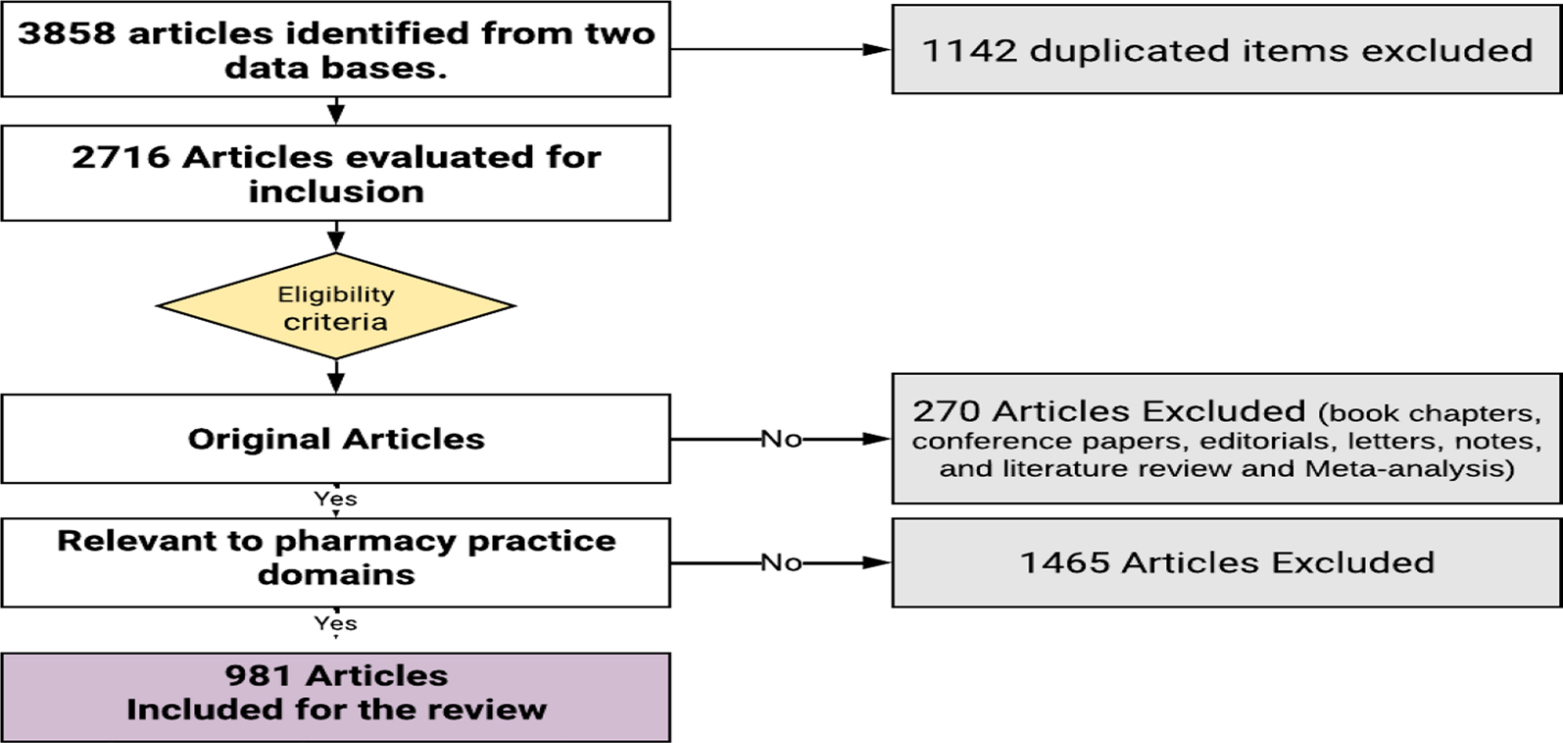
Study selection

A total of 3858 ‘hits’ were generated from the electronic search of both databases. 2521 and 1337 items were identified from Scopus and PubMed, respectively. 1142 of which were duplicates and omitted. Out of 2716 articles; 270 articles were classified as book chapters, conference papers, editorials, letters, notes, literature reviews, and Meta-analyses, thus did not meet the inclusion criteria and were excluded. Overall, 2446 articles were analyzed for inclusion, from which 981 were classified as pharmacy practice research papers in the selected countries and included in the review, as shown in Fig. 2.

### Included studies characteristics

Of the 981 reviewed articles, around 31% (*n* = 302) of studies were conducted in the KSA, followed by Jordan with almost 22% (*n* = 215) of the total included studies. Qatar comes in the third-place *n* = 114 articles, whereas *n* = 82 studies were conducted in the UAE.

The rest of the countries had fewer number of pharmacy practice research and can be arranged in descending order; Lebanon *n* = 71, Iraq *n* = 46, Kuwait *n* = 39, Yemen *n* = 35 and similarly in Palestine *n* = 33, Oman *n* = 22, Syria *n* = 9, and Bahrain *n* = 6.

A scarcity of pharmacy practice research articles was observed in Bahrain, only six articles were found for this review. One article was published in both 2009 and 2010, whereas two articles were in both 2012 and 2018. Only nine articles included in this review were conducted in Syria. One of them is interventional, while the rest are non-interventional. Twenty-two articles were recognized as “pharmacy practice research” in Oman. Yemen, and Palestine, despite the unstable political situation 35, 33 articles were noted in each, respectively. Whereas, 39 articles were affiliated in Kuwait; the majority of the articles were in the last 3 years of the decade 2017–2019. While 46 studies were conducted in Iraq and 71 in Lebanon.

KSA, Jordan, Qatar, and the UAE were the frontrunners in terms of the number of articles published in pharmacy practice. Coordination between different Middle East countries was observed in seven studies. One between Jordan and the United Arab of Emirates, the other in Qatar and Kuwait, two between Qatar and Lebanon. Table 2 demonstrates the percentage (Number) of articles identified in each country.

**Table 2:** Included articles in each country

Sr. no.:	Country name	% (*n*) of articles included
1	Bahrain	0.6% (6)
2	Iraq	4.7% (46)
3	Jordan	21.9% (215)
4	KSA	30.8% (302)
5	Kuwait	4.0% (39)
6	Lebanon	7.2% (71)
7	Oman	2.2% (22)
8	Palestine	3.4% (33)
9	Qatar	11.6% (114)
10	Syria	0.9% (9)
11	UAE	8.36% (82)
12	Yemen	3.6% (35)
13	More than one country	0.71% (7)
Total		981

**Fig. 3:**
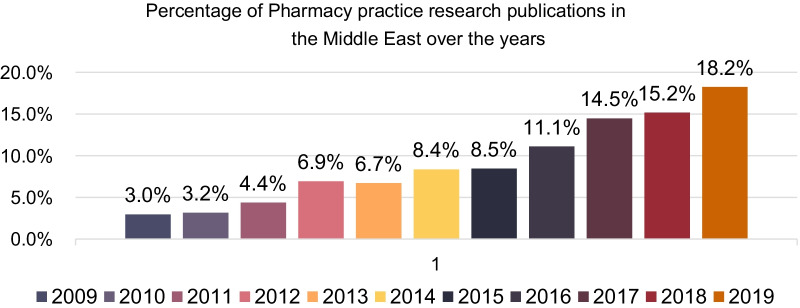
Articles distribution through the years

An increase in the number of publications was observed in the last 4 years, almost 15% increment of the publications in 2019, 18.25% (*n* = 179) compared to 2009 2.96% (*n* = 29). Table 3 and Fig. 3 present the included articles’ distribution through the years.

**Table 3:** Articles distribution through the years

Years	% (*n*) of articles
2009	2.96% (29)
2010	3.16% (31)
2011	4.38% (43)
2012	6.93% (68)
2013	6.73% (66)
2014	8.36% (82)
2015	8.46% (83)
2016	11.11% (109)
2017	14.48% (142)
2018	15.19% (149)
2019	18.25% (179)

The thematic analysis generated eight themes;

1. Theme 1: Pharmacy Practice and Pharmacist Services.
2. Theme 2: Medication Use and Pharmacogenomics.
3. Theme 3: Medication Safety and Pharmacovigilance.
4. Theme 4: Pharmacy Education and Professional Development.
5. Theme 5: Medicines Information and Public Health Promotion.
6. Theme 6: Pharmacoeconomics and Pharmaceutical Policies Studies.
7. Theme 7: Pharmaceutical Marketing Studies.
8. Theme 8: Clinical research.

The article classification among the themes is demonstrated in Tables 4. Table 5 shows the article distribution between countries per theme, while Table 6 shows the themes distribution per country.

**Table 4:** Articles classified in each Theme

Sr. no.	Theme		% (*n*) of articles included	
1	**Pharmacy practice and pharmacist services 22.94% (107)**		22.94% (225)	
	Community pharmacy	47.55% (107)		
	Hospital pharmacy	30.22% (68)		
	Clinical pharmacy	20.44% (46)		
	General pharmacy	1.78% (4)		
2	**Medication use and pharmacogenomics**		30.78% (302)	
3	**Medication safety and pharmacovigilance**		13.56% (133)	
4	**Pharmacy education and professional development**		16.31% (160)	
5	**Medicines information and public health promotion**		1.94% (19)	
6	**Pharmacoeconomics and pharmaceutical policies studies**		5.10% (50)	
7	**Pharmaceutical marketing studies**		2.75% (27)	
8	**Clinical research**		6.63% (65)	
Total			981

**Table 5:**
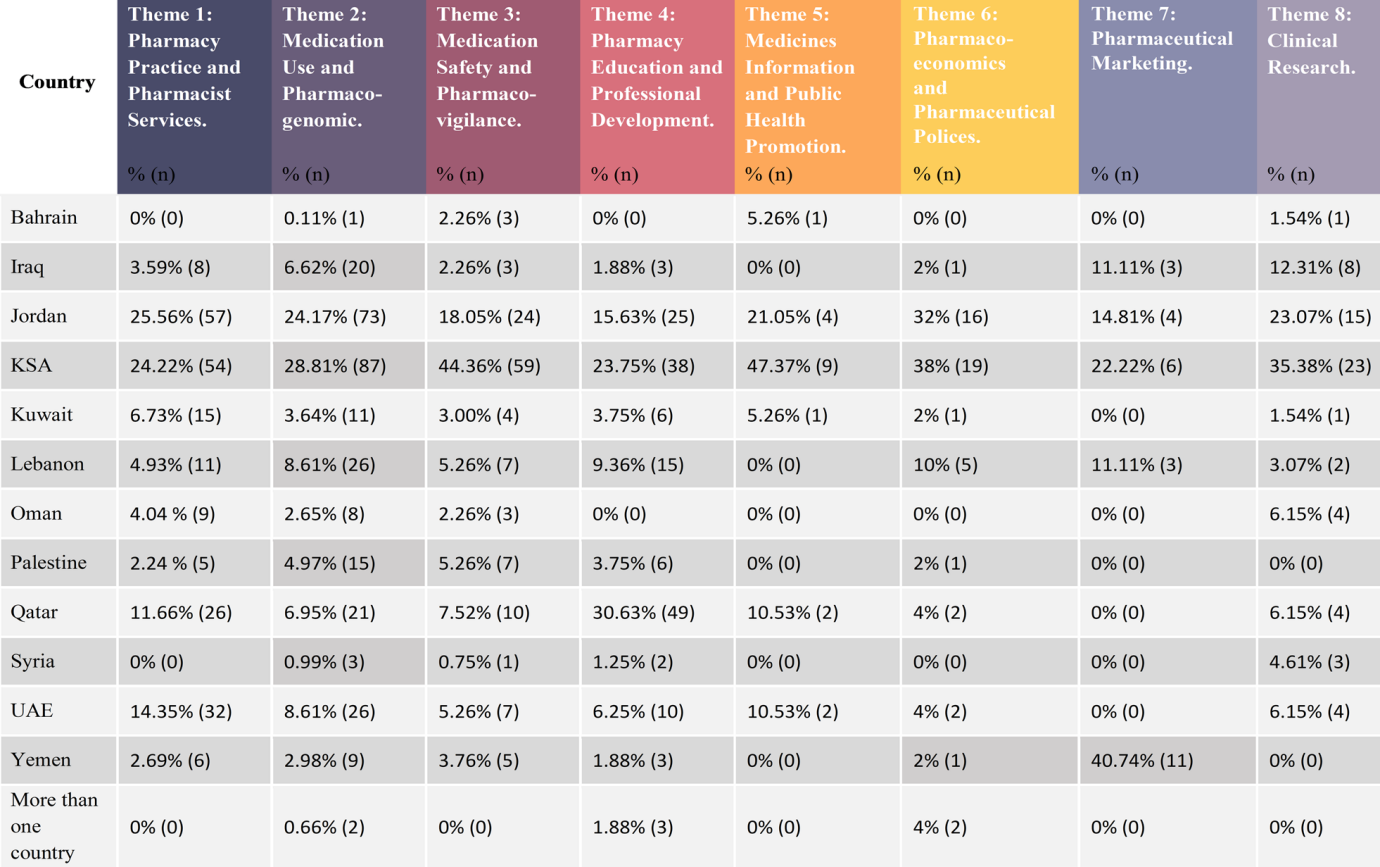
Articles distribution between countries per theme

**Table 6:**
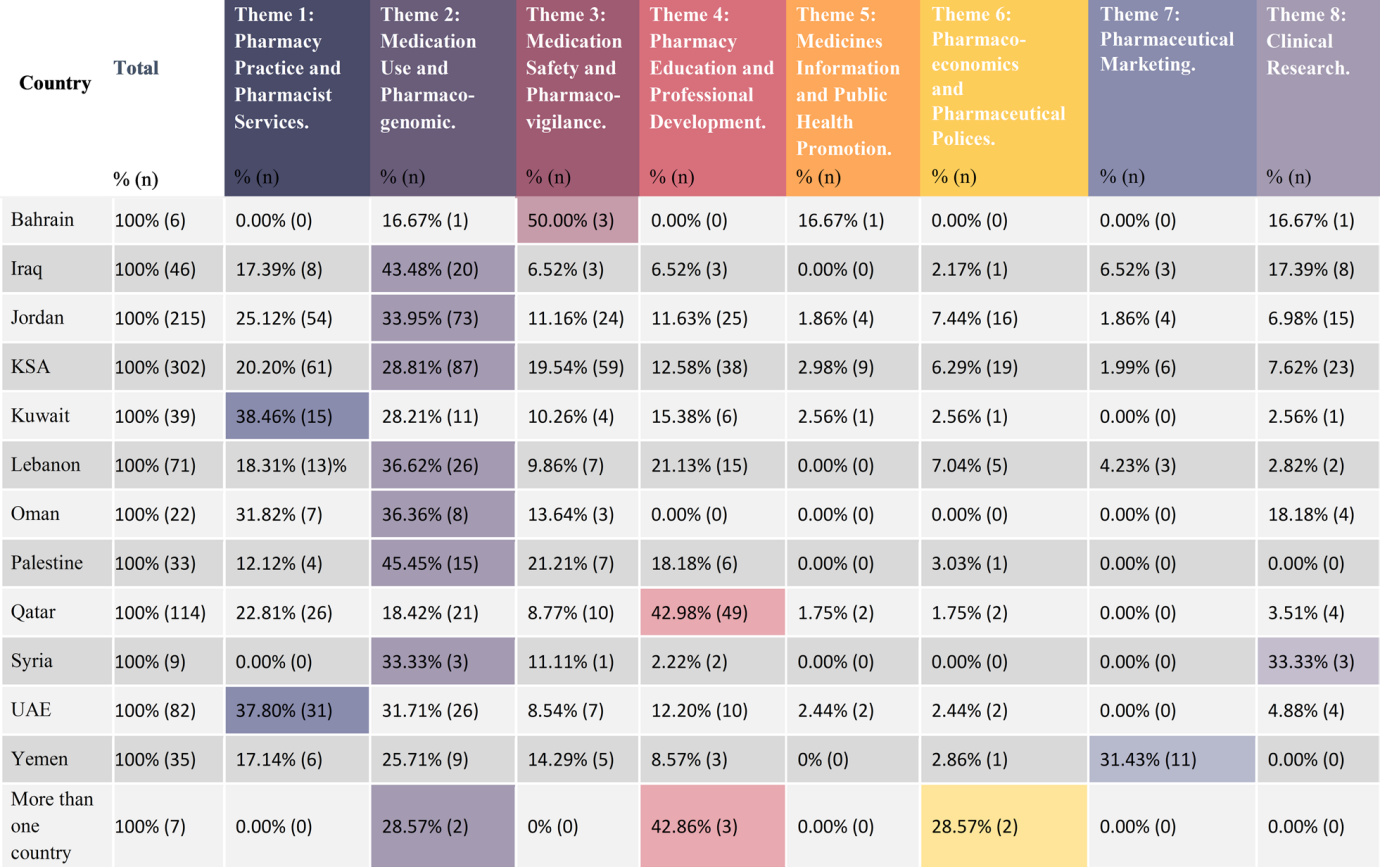
Themes distribution per country

The predominant theme was Theme 2: Medication Use and Pharmacogenomics which incorporated *n* = 302 papers. Approximately, one-quarter of the studies (22.94%) discussed the pharmacy practice and pharmacist services in different settings which were combined in theme 1; community pharmacy, hospital pharmacy, and clinical pharmacy.

One hundred and sixty articles were included in Theme 4 that covers the educational aspects of pharmacy in addition to the professional development concerns.

However, Theme 3: Medication Safety and pharmacovigilance included *n* = 133 studies. The last three themes have the lowest number of articles in ascending order; Theme 5: Medicines Information and Public health promotion *n* = 19, Theme 7: Pharmaceutical Marketing studies *n* = 27, Theme 6: Pharmacoeconomics and Pharmaceutical Policies *n* = 50, and Theme 8: Clinical Research *n* = 65.

Comparing the studies conducted in each country, it was noticed that the prominent theme in several countries was theme 2 “medication use”. Iraq, the KSA, Lebanon, Palestine, Syria, Oman, and Jordan were among these countries. Theme 1: Pharmacy Practice and Pharmacist Services was the second most common theme in the majority of these countries. However, Kuwait and the UAE were found to have Theme 1 as their dominant theme in the pharmacy practice research. In addition, it was found that Theme 4: Pharmacy Education and Professional Development is the most frequently published theme in Qatar. Table 6 presents the thematic classification of the articles in each country.

### Theme 1: Pharmacy Practice and Pharmacist Services

The research articles concerning pharmacy practice and pharmacist services were covering different pharmacy settings and were classified accordingly, as shown in Table 7.

- Community pharmacy
- Hospital pharmacy
- Clinical pharmacy

**Table 7:** Theme 1: pharmacy practice and pharmacist services

Sr. no.:	Sub-thematic heading	No. of articles	
1	**Pharmacy practice review**		
	General practice	4	6
	Community pharmacy	2	
2	**Management aspects**		
	Community pharmacy	9	12
	Hospital pharmacy	3	
3	**Pharmacy services assessment**		
	Community pharmacy	43	59
	Hospital pharmacy	13	
	Clinical pharmacy	3	
4	**Pharmacists’ Interventions evaluation**		
	Community pharmacy	8	47
	Hospital pharmacy	17	
	Clinical pharmacist	22	
5	**Implementing and developing pharmacy practice**		
	Hospital pharmacy	5	14
	Clinical pharmacy	9	
6	**Public perception and attitude to pharmacy practice**		
	Community pharmacy	26	32
	Hospital pharmacy	6	
7	Physician and HCP perception to pharmacy practice		
	Community pharmacy	2	17
	Hospital pharmacy	7	
	Clinical pharmacy	8	
8	**Pharmacist KAP to Pharmacist services**		
	Community pharmacy	15	23
	Hospital pharmacy	7	
	Clinical pharmacy	1	
9	**Physician–pharmacist collaboration**		
	Community pharmacy	2	15
	Hospital pharmacy	10	
	Clinical pharmacy	3	
Total		225	

Community pharmacy was the dominant setting in which pharmacy practice research studies were conducted (*n* = 107), this was followed by the hospital (*n* = 68) and clinical (*n* = 46) settings correspondingly.

Nine sub-thematic headings were observed and these are listed in Table 7.

i. Pharmacy practice review.
ii. Management aspects of pharmacy practice.
iii. Assessment of pharmacy services.
iv. Evaluation effectiveness of pharmacists’ interventions.
v. Implementing and developing pharmacy services.
vi. Public perception and attitude to pharmacy practice.
vii. Physician and Health Care Provider (HCP)s’ Perception to Pharmacy Practice.
viii. Pharmacists’ knowledge, attitude, and practice (KAP) to Pharmacist services.
ix. Physician–Pharmacist collaboration (multidisciplinary practice).

Six studies portrayed the pharmacy practice in general, two of these studies were about the community pharmacy. Regarding management aspects in pharmacy practice, nine studies were classified under this field in the community pharmacy. However, three studies assessed the management aspects of hospital pharmacy practice.

Forty-three studies assessed pharmacy services provided in community pharmacy settings and 13 studies were about hospital settings, only three articles discussed clinical pharmacy. Fourteen articles investigated the development and implementation of pharmacy services. However, none of this was about community pharmacy services, five were regarding hospital pharmacy, and 9 were about clinical pharmacy.

On the other hand, studies intended to evaluate the effectiveness of pharmacist intervention were mainly about clinical pharmacists *n* = 22 and hospital pharmacists *n* = 17 to a less extent about community pharmacists’ interventions *n* = 8.

Numerous studies highlighted the perceptions and attitudes of health care providers’ *n* = 17. Two concerning community pharmacy, seven concerning hospital pharmacy, and eight about clinical pharmacy. Pharmacists’ perceptions were observed in twenty-three studies *n* = 23: 15 were about community pharmacy, Thus, only seven about hospital pharmacy and one about clinical pharmacy. Public perceptions regarding pharmacy practice was observed in *n* = 32 studies: 26 regarding community pharmacy, and 6 regarding hospital pharmacy.

Inter-professional collaboration between the pharmacist and physician or in other words, multidisciplinary care, has gained increasing interest in the last decade *n* = 15. Two of these studies focused on the physician–community pharmacist collaboration. In addition, 10 studies were about the hospital pharmacists. Only three studies highlighted physician–clinical pharmacist collaboration.

### Theme 2: Medication Use and Pharmacogenomics 

The vast majority of the included studies shed the light on medication use aspects. Nearly, one-third (*n* = 302) of the total included articles were classed under the medication use themes. Each of the KSA and Jordan sheltered approximately 25% of the total articles under this theme (Table 5).

Five subheadings were identified under this theme:

1. Public behavior in regards to medication use.
2. Prescribing and dispensing practice.
3. Intervention to improve medication use.
4. Pharmacists and Health care providers' knowledge, attitude, and practice.
5. Pharmacogenomics studies.

The most utilized topic was the public behavior and practice in medication use (*n* = 116). A lack of studies assessing and discussing interventions used to enhance the medication use practice was observed *n* = 12. Table 8 shows the sub-themes of the articles.

**Table 8:** Theme 2: Mediation use

Sr. no.:	Sub-thematic heading	No. of articles
1	**Public behavior in regards to medication use**	
	Public medication use practice	116
	Self-medication practice and factors influence it	
	Medication adherence and factors affect it	
	Public KAP—medication use	
2	**Prescribing and dispensing practice**	
	Prescribing pattern	96
	Adherence to guidelines	
	Drug utilization pattern	
	Dispensing practice	
3	**Intervention to improve Medication use**	
	Intervention development	12
	Intervention evaluation	
4	**Pharmacist and HCP KAP**	
	Medication use—Pharmacist and HCP KAP	56
5	**Pharmacogenomics studies**	
	PG studies related to medication dosing	22
	KAP of HCPs to pharmacogenomics	
Total		302

### Theme 3: Medication Safety and Pharmacovigilance

Around 13% of the included articles were about medication safety, and two fifths (44.36%) of these articles were conducted in the KSA. Jordan was the second in regards to the number of articles published about medication safety (*n* = 24), while in Qatar, ten articles were published (Table 6).

1. Medication error:
  - Prevalence of medication error
  - Prescribing error
  - Dispensing error
  - Administration error
  - Medication error reporting
  - Interventions to prevent medication error
  - Factors affecting medication error.
2. Drug interaction
3. Drug-related problems—DRP
4. Medication safety—assessment
5. Medication safety knowledge and awareness
6. Pharmacovigilance studies

Studies depicting pharmacovigilance covered 36% of the articles followed by medication errors with almost one-third of the studies (*n* = 41) 30%. Table 9 demonstrates the sub-themes of the articles.

**Table 9:** Theme 3: medication safety and pharmacovigilance

Sr. no.:	Sub-thematic heading	No. of articles
1	**Medication error**	
	Prevalence of Medication error (General)	41
	Prescribing error	
	Dispensing error	
	Administration error	
	Medication error reporting	
	Interventions to prevent Medication error	
	Factors affecting Medication error	
2	**Drug interaction**	
	Medication safety—drug interactions	5
3	**Drug related problems**	
	Medication safety—DRP	22
4	**Medication Safety-assessment**	
	Assess Medication safety Problems in community pharmacy	2
5	**Medication Safety Knowledge and Awareness**	
	Public Awareness—counterfeit medicine	14
	Pharmacists’ and HCP.s’ KAP	
6	**Pharmacovigilance studies**	
	Review PV system	48
	Prevalence of ADR	
	Post-marketing surveillance	
	ADR Reporting assessment	
	Pharmacists’ and HCP.s’ KAP PV and ADR reporting	
	Public awareness of ADR	
Total		133

### Theme 4: Pharmacy Education and Professional Development

One hundred and sixty articles were identified and classified under the pharmacy education and professional development theme. Forty-nine papers were found in Qatar, while 38 studies were conducted in the KSA. However, no studies were found for Oman and Bahrain. Refer to Table 5.

Five sub-themes were identified including:

1. Curriculum and teaching strategies.
2. Pharmacy students’ perception toward pharmacy practice and pharmacy as a career.
3. Knowledge and competency evaluation.
4. Continuous education.
5. Research.

Below, Table 10 illustrates the sub-themes of the articles under theme 4: Pharmacy Education and Professional development.

**Table 10:** Theme 4: Pharmacy Education and Professional development

Sr. no.:	Sub-thematic heading	No. of articles
1	Curriculum and teaching strategies	
	Pharmacy Education Programs and curriculum Development	77
	Pharmacy Education Programs and curriculum Evaluation	
	Teaching methods assessment	
	Practical training	
	Pharmacy Students’ Perceptions of teaching methods	
	Pharmacist and Pharmacy students’ perception of Educational Programs vs. Practice	
2	Pharmacy Students’ perception	
	Pharmacy Students’ Perception of pharmacy as a career	18
	Pharmacy Students’ Perception of Pharmacy Practice	
3	Knowledge and Competency evaluation	
	Pharmacy and HCP Students’ Knowledge assessment	34
	Pharmacists’ and HCP.s’ Competency assessment	
4	Continuous education	
	Pharmacist and HCP Continuing Education	20
5	Research	
	Research regulations	11
	Perspective to research assessment	
	Implementing and assessing strategies to improve pharmacy practice research	
Total		160

### Theme 5: Medicines Information and Public health promotion

Medicines information resources and public health promotion aspects were investigated in 19 studies that either investigated the available and utilized medicines information resources *n* = 6, or evaluated the medicines Information and health promotion services provided by the pharmacists *n* = 7, in addition to six more studies examined public health awareness.

These studies were conducted in the Gulf Cooperation Council Countries; the KSA *n* = 9, the UAE *n* = 2, Qatar *n* = 2, and one in Kuwait, and Bahrain, in addition to Jordan *n* = 4 (Table 6). Table 11 illustrates the sub-theme classification of the articles under theme 5.

**Table 11:** Theme 5: medicines Information and Public health promotion

Sr. no.:	Sub-thematic heading	No. of articles
1	Medicines Information resources	6
2	The Medicines Information and health promotion services	7
3	Public Health Knowledge	6
Total		19

### Theme 6: Pharmacoeconomics and Pharmaceutical Policies Studies

Almost 5% of the included articles were classified as pharmacoeconomics and pharmaceuticals policies studies. The majority of these studies were conducted in the KSA followed by Jordan, Lebanon, UAE, and Qatar, respectively. In addition to two collaborative studies from Qatar and Lebanon (Tables 5 and 6). Table 12 illustrates the sub-thematic classification of the articles.

**Table 12:** Theme 6: Pharmacoeconomics and Pharmaceutical Policies Studies

Sr. no.:	Sub-thematic heading	No. of articles
2	Medication availability	8
3	Medication pricing	6
	Pharmaceutical financial policies	3
	Formulary decision making	8
4	Pharmacoeconomics evaluation studies	25
Total		50

### Theme 7: Pharmaceutical Marketing Studies

Pharmaceutical marketing was a prevalent topic in the literature and was covered by 27 papers. Yemen had the majority of these studies with 11 research papers followed by the KSA and Jordan with six and four studies, respectively, whereas Iraq and Lebanon had three studies each (Tables 5, 6).

Five sub-themes emerged from this theme. More than the half of included studies were about the physicians’ and/or pharmacists’ perception of the interaction between medication representatives and HCP 55% (15). Table 13 presents the sub-themes heading included in theme 7.

**Table 13:** Theme 7: Pharmaceutical Marketing Studies

Sr. no.:	Sub-thematic heading	No. of articles
1	Apprising the quality of drug promotions	4
2	Assess the influence of drug promotion on practice and prescribing behavior of healthcare providers	5
3	The interaction between medication representatives and HCP—physicians and/or pharmacists Perception	15
4	The interaction between medication representatives and HCP-Public Perception	2
5	Drug promotion technique	1
Total		27

### Theme 8: Clinical research

A limited number of clinical studies with a pharmacist’s role was observed in the literature *n* = 65. The majority of these studies were in the KSA *n* = 23 followed by Jordan *n* = 15 and Iraq *n* = 8. Fewer studies were observed in Qatar *n* = 4, Oman *n* = 4, the UAE *n* = 4, Syria *n* = 3, Lebanon *n* = 2, and Kuwait *n* = 1 (Tables 5 and 6).

Four domains were listed under this theme; Pharmacotherapeutic evaluation studies were the most frequently conducted studies in this theme *n* = 45, followed by Etiology and Risk Factor Studies *n* = 11, Clinical Pharmacokinetics studies *n* = 6, a Disease Prevalence Studies *n* = 3. Table 14 illuminates the sub-thematic classification of the articles.

**Table 14:** Theme 8: Clinical research

Sr. no.:	Sub-thematic heading	No. of articles included
1	Pharmacotherapeutic evaluation	45
2	Disease prevalence studies	3
3	Etiology and risk factor studies	11
4	Clinical pharmacokinetics studies	6
Total		65

## Discussion

This study highlighted the status of pharmacy practice research in the Middle East. KSA, Jordan, Qatar, and the UAE were the leaders in terms of the number of articles published in pharmacy practice, the KSA as the largest country amongst the included ones. The increasing number of published papers might be an influence of the Kingdom 2030 vision [28], that aims to enhance the development in different sectors including health, education, and economics and consequently lead to support the scientific research as well as pharmacy practice and health research. The UAE and Qatar are high income countries that place a great emphasis on scientific research [29]. Jordan is considered one of the key countries in pharmacy education and practice. This is with great number of pharmacy colleges, and advanced pharmacy practice policies. The unstable political situation in several Arab countries such as Lebanon, Iraq, Palestine, Yemen, and Syria may explain the less number of articles published in these countries.

An increasing number of publications was observed in the past 3 years that may show the trend towards academic institutions ranking and accreditation requirements from regulatory bodies [30]. This may also explain the increase in the number of publications.

Duplicated studies with repetitive ideas were observed throughout the studies analyzed, that may lead to saturation in certain fields and a gap may be observed in other fields.

### Pharmacy practice and pharmacist services

Regarding studies concerning pharmacy practice and pharmacist services, the bulk of studies was in community pharmacy settings; this can be elucidated by the popularity of community pharmacies [31]. Studies investigating the developing and implementing pharmacy services are needed in all pharmacy settings, especially in community pharmacies. A bibliometric study published in 2014 has also indicated the negligence of this field in the Arab world [32].

### Medication use

Medication use is included in many research articles, and the most frequent topic is the use of antibiotics and antimicrobial stewardship programmes [33]. Socio-behavioral aspects in medication use and specially self-medication was a common area of focus from different authors. An expected reason might be the importance of assessing self-medication and ensuring its appropriateness the need for law reinforcement to manage the medication dispensing and distribution. A systematic review in the Middle East about the self-medication also supports this recommendation [34] and enhances the public awareness toward this issue. A limited number of studies discussed the interventions and solutions to improve medication use. A systematic review about the safety of medication use in the GCC also supports this view [35].

### Medication safety

Medication safety was the fourth most utilized topic, and pharmacovigilance related studies covered almost 40% of the literature. This is due to the fact that pharmacovigilance is still considered in its infancy in several Arab and Middle East countries [24, 35].

Medication error was the second most topic in the field of medication safety. The majority of studies were regarding the prevalence and factors affecting this practice. However, there was a shortage of studies that assess and evaluate the interventions to prevent and improve pharmacy practice in this regard. This is in line with a systematic review published in 2013 about medication errors in the Middle East [23].

### Pharmacy education

Qatar was the first in studies concerning pharmacy education programmes and curriculum development and evaluation, followed by the KSA and Jordan.

Conversely, Qatar has a single higher educational institute that offers a pharmacy degree that has been launched in 2007 [36], but pronounced support for pharmacy education is noticed in the fast growth. The KSA pharmacy education dates back to 1959 with only one college and after that in the first decade of the second centenary, 23 government colleges, and seven private colleges had been lunched [37], as per the descriptive study conducted in 2018, this increase in educational institutes raise the demand of research in the pharmacy education field [38]. Jordan has the second oldest pharmacy programme in the region starting from 1979, and today several pharmacy schools offer bachelor’s, master’s, and doctorate programs. This may explain the huge number of publications in Jordan [39]. The establishment of the first pharmacy college in the UAE was in 1992, afterward, numerous pharmacy programs were established [40]. The maturity of the pharmacy education programme has played an affirmative role in enhancing the pharmacy practice research in these countries. This is in addition to the strong economics as well as a political stability.

Related to other topics a scarcity of studies about continuous education for pharmacists and health care providers *n* = 20 and scientific research (*n* = 11) was identified. High interest in scientific research was observed in Qatar and Jordan compared to the other countries.

The most commonly utilized method in studies was to assess the knowledge and perception through quantitative questionnaire-based studies. The least used methods were simulated patient or mystery shopper-based qualitative methods.

In agreement with the bibliometric review pharmacy education literature published in 2013, several gaps in the literature were outlined to develop a research agenda in the region [41].

### Pharmacoeconomics and pharmaceutical marketing research

KSA, Jordan, Yemen, Lebanon, and Iraq, respectively, had the greatest number of studies in pharmacoeconomics and pharmaceutical marketing. According to the United Nations World Bank; the KSA is considered a high-income country, thus Jordan, Lebanon, and Iraq from the upper-middle-income countries. However, Yemen is among the low-income countries [42–44]. It is justified why middle and low-income countries emphasize pharmacoeconomic research studies as it will help in the allocation of inadequate health resources [45].

Pharmaceutical marketing was a dominant topic in Yemen, this might be due to the lack of regulations that govern the relationship between the pharmaceutical industry and the health care providers as elucidated in a review article discussing the pharmacy practice challenges in Yemen [46].

## Conclusions

Most studies were conducted in the KSA, followed by Jordan, Qatar, and the UAE. Syria and Bahrain have comparatively a smaller number of studies being conducted. Coordination between Jordan, the United Arab of Emirates, Qatar Kuwait, and Lebanon was observed in several articles. The prominent themes were medicines use, pharmacy practice and pharmacist services, pharmacy education and professional development, and clinical research. It was concluded that the published pharmacy practice research in the included countries have a similarity of themes, and some ideas were repetitive. There is a need to develop a research agenda based on the findings. This would be helpful to enhance the status of the pharmacy practice research in the Middle East.

### Recommendations

The recommendations coming from this work are separately published [47]. These include identifying research gaps, research collaboration between academic researchers and practitioners, and focusing on applied, interventional, and implementational research [47].

## Data Availability

The data that support the findings of this study are available from the corresponding author upon reasonable request.
